# Active aging – resilience and external support as modifiers of the disablement outcome: AGNES cohort study protocol

**DOI:** 10.1186/s12889-018-5487-5

**Published:** 2018-05-02

**Authors:** Taina Rantanen, Milla Saajanaho, Laura Karavirta, Sini Siltanen, Merja Rantakokko, Anne Viljanen, Timo Rantalainen, Katja Pynnönen, Anu Karvonen, Inna Lisko, Lotta Palmberg, Johanna Eronen, Eeva-Maija Palonen, Timo Hinrichs, Markku Kauppinen, Katja Kokko, Erja Portegijs

**Affiliations:** 1 Gerontology Research Center, Faculty of Sport and Health Sciences, Univerisity of Jyvaskyla, P.O. Box 35 (viv 149), 40014 Jyväskylä, Finland; 2 Division of Sports and Exercise Medicine, Department of Sport, Exercise and Health, University of Basel, Basel, Switzerland

**Keywords:** Aging, Wellbeing, Disability, Activity, Environment, Mobility, Exercise, Functioning, Lower extremity, Cohort

## Abstract

**Background:**

Population aging increases the need for knowledge on positive aspects of aging, and contributions of older people to their own wellbeing and that of others. We defined active aging as an individual’s striving for elements of wellbeing with activities as per their goals, abilities and opportunities. This study examines associations of health, health behaviors, health literacy and functional abilities, environmental and social support with active aging and wellbeing. We will develop and validate assessment methods for physical activity and physical resilience suitable for research on older people, and examine their associations with active aging and wellbeing. We will examine cohort effects on functional phenotypes underlying active aging and disability.

**Methods:**

For this population-based study, we plan to recruit 1000 participants aged 75, 80 or 85 years living in central Finland, by drawing personal details from the population register. Participants are interviewed on active aging, wellbeing, disability, environmental and social support, mobility, health behavior and health literacy. Physical activity and heart rate are monitored for 7 days with wearable sensors. Functional tests include hearing, vision, muscle strength, reaction time, exercise tolerance, mobility, and cognitive performance. Clinical examination by a nurse and physician includes an electrocardiogram, tests of blood pressure, orthostatic regulation, arterial stiffness, and lung function, as well as a review of chronic and acute conditions and prescribed medications. C-reactive protein, small blood count, cholesterol and vitamin D are analyzed from blood samples. Associations of factors potentially underlying active aging and wellbeing will be studied using multivariate methods. Cohort effects will be studied by comparing test results of physical and cognitive functioning with results of a cohort examined in 1989–90.

**Conclusions:**

The current study will renew research on positive gerontology through the novel approach to active aging and by suggesting new biomarkers of resilience and active aging. Therefore, high interdisciplinary impact is expected. This cross-sectional study will not provide knowledge on temporal order of events or causality, but an innovative cross-sectional dataset provides opportunities for emergence of novel creative hypotheses and theories.

## Background

In countries with aging populations, a key question regarding quality of life, sustainability, productivity, and equity of societies concerns whether old age is lived with functional limitations, disability and limited opportunities to contribute to the society, or whether it is spent with opportunities for participation and involvement in meaningful life situations. During the recent years, the idea of active aging has been essential in positive gerontology, in innovation programs and in policy. The concept of active aging stems from the work of Robert Havighurst who launched the activity theory [1]. The activity theory posits that staying active in later life will lead to maintenance of wellbeing. The contributions of older persons to their own wellbeing are of focal interest in many countries with growing proportion of older people in the population. Signaling this, World Health Organization defined the policy goal of active aging in 2002 as follows: “Active aging is the process of optimizing opportunities for health, participation and security in order to enhance quality of life as people age” [2]. The same document also states, “these policies and programs should be based on the rights, needs, preferences and capacities of older people”. The European Union programs such as the European Innovation Partnership on Active and Healthy Aging further demonstrate the importance of active aging in policy and innovation [3].

In gerontology, it is necessary to realize the distinction between active aging policies and active aging of individuals. Policies are goal-oriented actions of authorities while active aging in individual lives may not be categorized solely based on policy goals. Even though there has been a lot of attention on active aging, the empirical research and knowledge of active aging has progressed only little, and its definition and content have remained varied. To this end, we developed the following definition of active aging that is centered on the individual: “The striving for elements of wellbeing through activities relating to a person’s goals, functional capacities and opportunities” [4]. We developed a scale to assess active aging of individuals that is entitled the University of Jyvaskyla Active Aging Scale (UJACAS) [4]. This scale includes the four central sides in the active aging of individuals: their goals (what they want to do), their functional capacity (what they are able to do), their autonomy (perceived opportunities to do the valued activities) and their activities (what they actually do). We are not aiming to define active aging as a static position with clear-cut boundaries. Instead, we try to capture it as the striving for elements of wellbeing through activities relating to a person’s goals, functional capacities and opportunities.

Changes in self-reported disability, a reflection of an individual’s abilities relative to environmental requirements, do not necessarily reflect changes in the physical functioning of individuals but may result from changes in the environment. Muscle strength, reaction speed or sensory functions are the building blocks of functioning and active aging in terms that they underlie mobility (e.g. [5]), activities of daily living [5–7] and opportunities to take part in social life [8]. In Finland, the cohort comparisons among 65-year-old people suggest that socioeconomic situation has improved, physical activity is more common and self-ratings of health and functioning have improved but prevalence of chronic conditions has not changed [9]. Another study in Finland among 90-year-olds suggests that disability prevalence is increasing in oldest old cohorts [10]. However, among Danish nonagenarians cognitive functioning and activities of daily living functioning had improved [11]. In a Swedish study among 75-year-olds, gait speed had increased in later born cohorts, which was explained by taller body height in a more recent cohort [12].

Aging increases the risk of comorbidity and decline in the body functions and structures, which compromises the opportunities for active aging. Resilience refers to the ability to resist or recover from adverse effects of a stressor and it comes to play when a risk is present [13]. Earlier research has predominantly concentrated on social and psychological resilience and physiological indicators of resilience have been less studied. Physiological and psychological functions show great plasticity in old age and respond positively to training and rehabilitation and negatively to inactivity and disuse. Resilience is particularly salient for aging as the risk of stressors increases and the capacity to recover from them diminishes. Potential biomarkers of resilience are heart rate and heart rate variability since changes in heart rate reflect the function of the autonomic nervous system.

Active aging encompasses the idea that in case of functional limitations, active aging may be reached by compensating for the reduced abilities by environmental or social support. Addressing the person-environment interplay with objective technologies based on geographical maps and by recording physical activity and associated individual physiological responses under everyday living conditions and in standardized laboratory conditions with wearable sensors will provide novel knowledge on the person-environment interactions expanding knowledge that is based on reported perceptions of individuals. Social engagement refers to being part of a supportive social group and having various supportive resources, such as emotional, informational, finances or companionship. Social engagement is a resource for active aging.

**Fig. 1:**
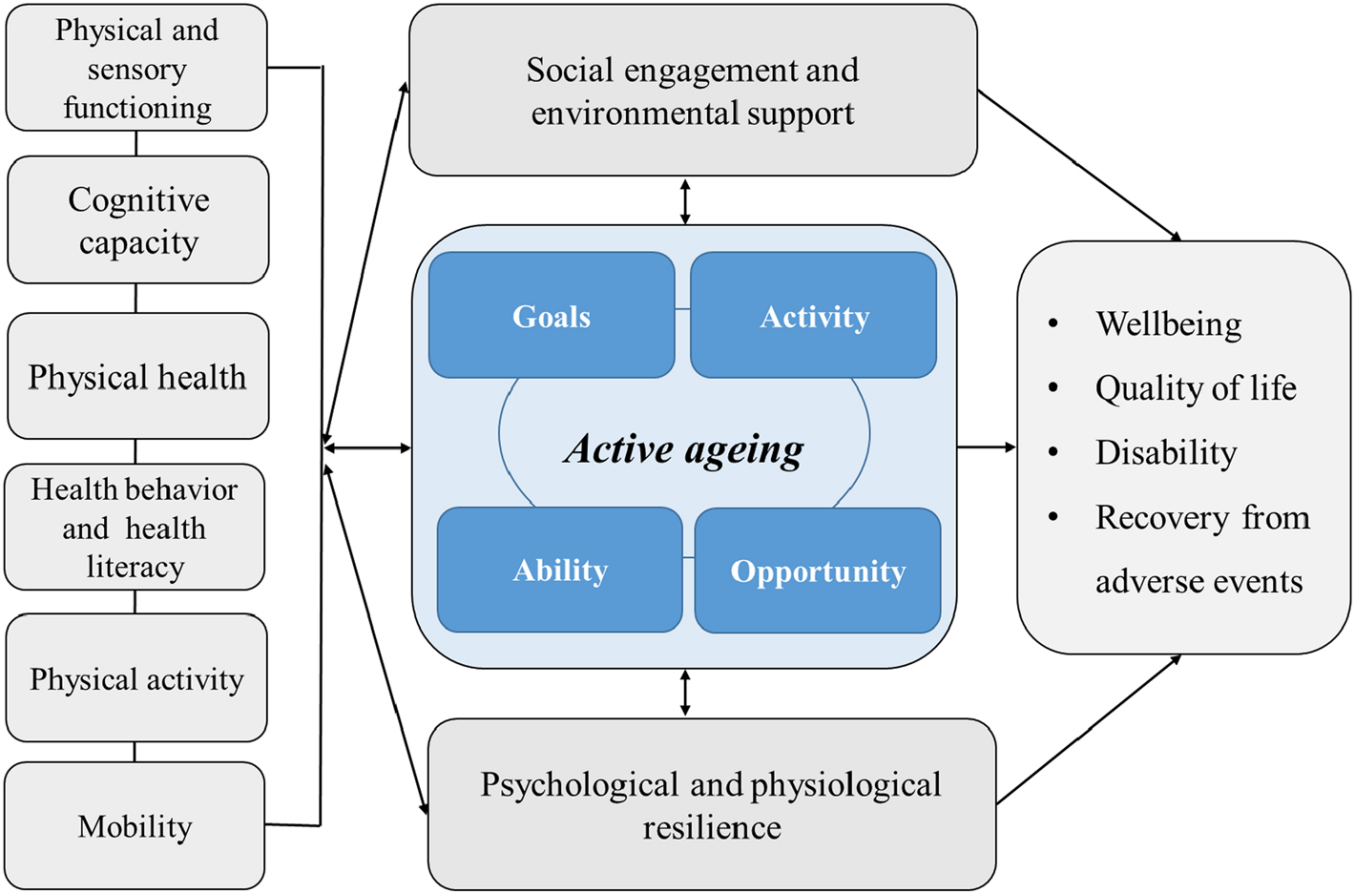
The conceptual framework of the current study. Active aging refers to the activity as per one’s goals, opportunities and abilities. It mediates or modulates the association of health and functioning with disability and wellbeing, while social engagement, environmental support and resilience influence this process, especially when facing adverse events

Figure 1 shows the conceptual framework of the current study. The current study will further the research on active aging by addressing the individual and environmental factors relating to active aging and by assessing the benefits of active aging for the individuals. The current study will also produce new knowledge on whether the more recent cohorts of older people have better functional abilities than earlier cohorts.

### Project aims

1. To examine health, health behaviors, health literacy and functional abilities as predictors of active aging.
2. To examine if active aging supports wellbeing in old age (even) when facing functional decline.
3. To examine how environmental support and social engagement influence active aging and wellbeing.
4. To study the person-environment interactions underlying active aging and disability.
5. To develop and validate objective assessment methods for physical activity suitable for research on older people, and examine their associations with active aging and wellbeing.
6. To develop and validate biomarkers of physical resilience suitable for research on older people, and examine their associations with active aging and wellbeing.
7. To study cohort effects on functional phenotypes that underlie active aging and disability.

## Methods

### Study design

AGNES is an observational study of three age cohorts (75, 80, and 85 years) with ongoing cross-sectional data collection (including phone and face-to-face interviews, postal questionnaire and assessments in a research center) and one-week activity surveillance with wearable monitors. In addition, the data will function as a baseline for an intervention study promoting active aging (trial registration number ISRCTN16172390) and it may function as a baseline for a future longitudinal study of the cohorts. The data collection is partly harmonized with a previous cohort study (Evergreen) including 75- and 80-year-old people assessed in 1989–1990 [14], thus providing an opportunity for cohort comparison.

### Sample size and power calculations

In the cross-sectional analysis for continuous variables a sample size of 1000 yields a power of approximately 99% to show a contribution to the explained variance of 5% in a linear regression model with 10 predictors (including interactions, but not constant) if the probability level (alpha) is set at 0.05. A sample size of 1000 yields weak correlation coefficients (*r* = 0.1) statistically significant. For cohort comparisons the sample size of 650 will give us 80% power to observe a 5% difference in continuous variables between the cohorts if the standard deviation is approximately a third of the mean as indicated by pilot data.

### Recruitment process and inclusion criteria

The population register is used to target 75-, 80- and 85-year old people living in Jyväskylä, Finland, in postal areas within a 10 kilometer radius of the city center or within reach of local public transportation. The population-based study is targeted to include 1000 participants in total. Based on an estimated participation rate of 50% in previous studies, a sample of at least 2000 people would be needed. Participants aged 75 years old are recruited since September 2017, and in 2018, those aged 80 years and 85 years will be recruited. Samples are drawn from population registers shortly before the initiation of the assessment of each age-cohort. Inclusion criteria for the population-based study are age 75, 80 or 85 years, and living independently in the recruitment area (Jyväskylä). Exclusion criteria are unwillingness to participate and inability to communicate.

### Contacting participants

**Fig. 2:**
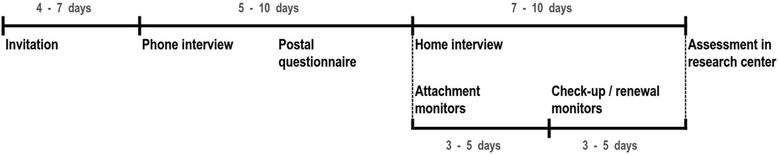
Approximate timeline of participant contacts (indicated with vertical lines) and data collection from participants

Participants are invited to participate in the study by a letter informing them of the cohort study. Within a week a trained interviewer contacts them by phone to assess their willingness to participate. If they agree to participate, a home visit is scheduled and a reminder and a postal questionnaire are sent to them by mail. At the beginning of the home visit, the participant signs a written informed consent form. Following the home interview, participants are invited to participate in the physical activity surveillance (including an additional home visit to check and renew monitors) and the functional assessments in the research center. Figure 2 shows the approximate timeline of participant contacts.

### Data collection

#### Phone interview

During the initial phone interview, eligibility of potential participants is screened. A brief non-respondent interview is conducted, including five selected questions from the home interview on health and functioning. In addition, reasons for non-participation are asked from those who do not wish to participate in the study. If an individual agrees to participate, a time and date for the home interview is scheduled.

#### Postal questionnaire

Participants, for whom a home interview is scheduled, are asked to complete a questionnaire prior to the home interview. The interviewer returns the completed questionnaire to the research center or the participant brings the completed form to the research center at the time of the functional assessments.

#### Home interview

A face-to-face interview in the participants’ home is conducted by trained research staff to collect data on activity, health, functioning, and wellbeing. In addition, two short performance tests are conducted. Home interviews enable those with poorer health and function to participate.

#### Physical activity surveillance

Immediately following the home interview, participants are asked for their willingness to participate in the physical activity surveillance. Data on participants’ physical activity are collected using accelerometers and single-channel electrocardiogram (ECG) recorders. Participants are instructed to wear these devices day and night for 7 to 10 days, preferably until the assessments in the research center. While wearing these devices, participants complete a short diary on their daily activities. Halfway the assessment period, the interviewer renews the attachments and replaces the ECG recorder. Participants with a pacemaker are excluded from using an ECG recorder. As the activity monitors are not fully water-resistant, participants swimming, bathing or taking a sauna multiple times per week, and who are not willing to reduce these activities, are excluded from participation in the surveillance altogether.

#### Assessment in the research center

In the research center of the Faculty of Sport and Health Sciences of the University of Jyvaskyla, Finland, data are collected using a clinical examination, physical and cognitive performance tests, interviews, and physical activity monitors.

Participants receive written and verbal instructions for preparation of the assessments in the research center during the home interview. Participants are asked to refrain from extreme physical exertion, staying up late, and drinking alcohol on the day before the assessments, and not to consume caffeine containing drinks and not to smoke during the 2 h preceding the assessments. In addition, they are advised to consume only light meals on the day of the assessments, preferably no less than 2 h before arrival. Following the clinical examination and the anthropometric assessments, participants are provided with a light meal and drinks without caffeine.

#### Objective environmental data

Objective data on physical environmental features of the home environment of participants will be extracted from freely available map-based (geographical) resources. Where existing data quality is insufficient, these data will be supplemented by newly collected data using virtual audits (e.g. Google Streetview). The collection of environmental data does not require any involvement of participants.

### Ethics

The ethical committee of the Central Finland Health Care District provided an ethical statement on the AGNES study protocol on August 23, 2017. The study includes no invasive or potentially physically or psychologically harmful elements beyond what one might experience in everyday life. The interviewer asks participants to sign a written informed consent form at the beginning of the home interview. The consent form contains separate boxes for the home interview, the assessments in the research center, utilization of the patient records, and permission to be contacted again for future satellite studies initiated before 2023. Patient records from public health care utilization are used only to confirm whether participants scoring 24 or lower on the Mini-Mental State examination are treated for cognitive problems, or whether they need to be referred to a health center for a check-up. During any contact with research staff, participants have the opportunity to ask for information about the study and its procedures. Participants are allowed to withdraw their consent at any time during the study or for any individual part of the study. For the duration of the home interview, assessments in the research center and immediately related travels, participants and research staff are covered by insurance for harm caused by accidents.

The data collection of objective environmental data (map-based or virtual audit) does not engage participants. To ensure participants’ privacy, a project researcher will identify virtual audit areas (aggregated for multiple participants) without revealing the exact participant addresses to assessors and link the participant data with the objective environmental data in a geographic information system (GIS).

In case of future studies or major protocol changes, a new ethical statement will be obtained and new informed consent procedures will be initiated for participants. The AGNES study follows the principles of the Declaration of Helsinki.

### Main outcomes

The main outcomes of this study are active aging and wellbeing and the secondary outcomes are resilience, physical activity, mobility, disability, environmental support and social engagement. For cohort comparisons, the main outcomes are physical, sensory and cognitive function, and the secondary outcome measures are wellbeing, health behavior, and physical activity.

### Measures

Finnish language is used for all questionnaires, interviews and assessments. Table 1 lists all measures of the study, indicates the data collection method, and whether the measure is available in the Evergreen cohort (collected in1989–90) and thus usable for cohort comparison.

**Table 1:** Descriptions, points of data collection, and references of the measures included in the study

Assessment/method/scale	P	H	R	S	C	Reference
Active aging						
Active Aging (68 items UJACAS Scale)	–	x	–	–	–	[4]
Perceived active aging (single item)	–	x	–	–	–	[4]
Motivation for active aging (single item)	–	x	–	–	–	[4]
Barriers to active aging (two items)	–	x	–	–	–	[4]
Perceived age (eight items)	–	x	–	–	–	[15]
Wellbeing and quality of life						
Psychological wellbeing (42-item Scales of Psychological wellbeing)	–	x	–	–	–	[18]
Emotional wellbeing (five-item Satisfaction with Life Scale)	x	–	–	–	–	[19]
Depressive symptoms (CES-D questionnaire; 20 items)	–	x	–	–	x	[21]
Quality of life (13-item OPQOL-brief questionnaire)	–	x	–	–	–	[24]
Perceived quality of life and life satisfaction (two items)	x	–	–	–	x	
Self-rated health (single item)	x	x	–	–	x	[9]
Perceived stress in life (single item)	–	x	–	–	x	
Sense of autonomy outdoors (five-item subscale of IPA)	–	x	–	–	–	[27]
Self-reported functional status (ADL & IADL) (twelve items)	x	–	–	–	–	[27]
Physical activity						
One-week physical activity surveillance (with tri-axial accelerometers and wearable ECG recorder)	–	–	–	x	–	[28]
Self-reported habitual physical activity level (single question)	–	x	–	–	x	[34]
Self-reported habitual physical activity (eight-item YAPS)	–	x	–	–	–	[35]
Unmet physical activity need (three items)	x	–	–	–	–	Adapted from [39]
Resilience						
Psychological resilience (ten-item Connor–Davidson Resilience Scale)	–	x	–	–	–	[40]
Physiological resilience (one-week heart rate and heart rate variability monitoring with wearable ECG recorder)	–	–	–	x	–	Adapted from [41]
Objective fatigability in cognitive and walking tests (heart rate, lactate, slowing)	–	–	x	–	–	Adapted from [45, 47, 48]
Subjective fatigability in cognitive and walking tests (repeated assessment of two items)	–	–	x	–	–	[43], adapted from [44]
Fatigability in daily life (13-item Situational Fatigue Scale)	–	x	–	–	–	[49]
Environmental support and social engagement						
Housing and living situation (four items)	–	x	–	–	x	
Perceived safety of the neighborhood (three items)	x	–	–	–	x	
Use and perception of neighborhood environment (SoftGIS)	–	–	x	–	–	Adapted from [50, 110]
Activity-space (SoftGIS)	–	–	x	–	–	Adapted from [51]
Objectively assessed environment (GIS)	–	–	–	–	–	Adapted from [52, 53]
Socioeconomic status (five items)	–	x	–	–	x	[54, 55]
Loneliness (single item)	x	–	–	–	x	[56]
Marital status (single item)	–	x	–	–	x	
Social contacts (four items)	x	–	–	–	x	[27]
Provision of social support (two items)	x	–	–	–	–	
Personal hobbies (single item)	–	x	–	–	–	
Perceived age discrimination (single item)	–	x	–	–	–	Adapted from [57]
Physical and sensory function						
Maximal isometric handgrip strength (hand-held dynamometer)	–	x	–	–	–	[58]
Maximal isometric strength of handgrip and knee extension (adjustable dynamometer fixed to a chair)	–	–	x	–	x	[34]
Reaction time and sensory motor speed (simple and complex task)	–	–	x	–	x	[59]
Lower extremity performance (SPPB)	–	x	–	–	–	[60, 61]
Ten-meter walking speed at habitual speed	–	–	x	–	–	[63]
Ten-meter walking speed at maximal speed	–	–	x	–	x	[64]
Six-minute walk	–	–	x	–	–	Adapted from [47, 66]
Gait characteristics (accelerometry)	–	–	x	x	–	[67, 68]
Self-reported hearing (three items)	–	x	–	–	x	
Objective hearing acuity (pure-tone audiometry)	–	–	x	–	–	[71]
Self-reported vision (three items)	–	x	–	–	x	
Binocular visual acuity (illuminated Landolt ring chart)	–	–	x	–	x	[72]
Respiratory function (spirometry)	–	–	x	–	x	
Cognitive capacity						
Cognitive impairment (19-item MMSE)	–	x	–	–	–	[74]
Trail Making Test	–	–	x	–	–	[75]
Digit span (item from Wechsler Memory Scale)	–	–	x	–	x	[81]
Digit symbol test (item from Wechsler Adult Intelligence Scale)	–	–	x	–	x	[84]
Phenomic verbal fluency test (item from Schaie-Thurstone Adult Mental Abilities Test)	–	–	x	–	x	[82]
Physical health						
Self-reported physician diagnosed diseases	–	x	–	–	–	Adapted from [27]
Severe pain, recent fractures and hospital admissions	–	x	–	–	–	
Prescription medication (self-reported)	x	–	–	–	–	
Clinical health examination	–	–	x	–	–	
Blood sample (total, HDL and LDL cholesterol)	–	–	x	–	x	
Blood sample (C-reactive protein, small blood count, and vitamin D)	–	–	x	–	–	
Resting electrocardiogram (ECG)	–	–	x	–	x	
Orthostatic test	–	–	x	–	x	[90]
Blood pressure and arterial stiffness (pulse wave analysis)	–	–	x	–	–	[91]
Body weight and height	–	–	x	–	x	
Body composition (bioimpedance, waist circumference)	–	–	x	–	–	
Physical frailty phenotype (five items)	x	x	–	–	–	[98]
Health behavior and health literacy						
Self-reported use of alcohol (four items)	x	–	–	–	–	
Self-reported smoking history (single items)	x	–	–	–	x	
Perceived own role in health behavior (three items)	x	–	–	–	x	
Nutritional status and habits (eight-item Abbreviated Screen II)	x	–	–	–	–	[99]
Health literacy (16-item HLS-EU-Q16 questionnaire)	–	x	–	–	–	[102]
Mobility						
Life-space mobility (15-item LSA questionnaire)	–	x	–	–	–	[105, 106]
Transportation modes (four items)	x	–	–	–	–	[111]
Self-reported mobility limitation (three items)	–	x	–	–	–	[107, 108]
Self-reported mobility task accommodation (three items)	–	x	–	–	–	[107]
Assistive device use (eight items)	–	x	–	–	–	[27]
Fear of falling (single item)	x	–	–	–	–	[27]
Fall history (three items)	x	–	–	–	–	
Other						
Debriefing (two items)	–	x	x	–	–	
Age and sex	–	–	–	–	x	
Life years	–	–	–	–	x	[9]

For each assessment, the point of data collection is indicated: part of the postal questionnaire (P), part of the home interview (H), part of the assessment in the research center (R), and part of the activity surveillance (S). In addition, availability of the measure in the historic cohort is indicated (C)

#### Active aging

Active aging is assessed using the University of Jyvaskyla Active Aging Scale (UJACAS; [4]), which includes the following 17 items: practicing memory, using computer, advancing matters in own life, exercising, enjoying the outdoors, taking care of appearance, crafting or DIY, making home cozy and pleasant, helping others, maintaining friendships, getting to know new people, balancing personal economics, making one’s days interesting, practicing artistic hobbies, participating in events, advancing societal/communal matters, and doing things according to one’s world view. Participants are asked to consider the previous 4 weeks. All items are assessed for the aspects of goals, ability, opportunity and activity and response options are worded to suit the item and scored from zero (lowest e.g. least active) to four (highest e.g. most active). Scores are computed by summing the scores of the individual items to form a subscale of each dimension (range 0–68) and a total score (range 0–272), when there are at most two missing items for the respective subscale or eight for the total scale. The scale has been shown to be valid and to have good test-retest reproducibility [4].

In addition, perceived active aging is assessed using a single question by asking participants to evaluate how active their life is on a scale from 0 to 10. To evaluate motivation for active aging, participants are asked how strongly they agree or disagree with the claim “I have special interests in my life”, rated on a five-point scale from one (strongly agree) to five (strongly disagree). In addition, perceived barriers to active aging are assessed by asking participants to what extent their a) health or functional ability or b) other matters related to their life or environment have prevented them from doing wanted activities during the previous 4 weeks. Responses were rated on a five-point scale ranging from one (very much) to five (not at all) [4].

Perceived age indicates a person’s experience on their own aging at a personal level. It is assessed with the following questions: “How old do you feel you are?”, “What age do you think you look like?”, “Do you feel mentally younger, as old as, or older than your chronological age?”, “Do you feel physically younger, as old as, or older than your chronological age?”, “How old would you like to be?”, “Have you felt age weighing on you?” (if yes, “At what age you began to feel that way?”), “At what age do you think old age begins?”, and “Would you like to live to be 100 years” [15].

#### Wellbeing and quality of life

Psychological wellbeing is assessed using the 42-item version of the Scales of Psychological Well-Being [16]. The 42-item version of the scale enables valid measurements of each of the components separately, yet minimizing respondent burden in comparison with the full 84-item version [17, 18]. The scales consist of six components (including seven items each), that is, autonomy (e.g., “My decisions are not usually influenced by what everyone else is doing”), environmental mastery (e.g., “In general, I feel I am in charge of the situation in which I live”), personal growth (e.g., “For me, life has been a continuous process of learning, changing, and growth”), positive relations with others (e.g., “Maintaining close relationships has been difficult and frustrating for me”; reverse scored), purpose in life (e.g., “I have a sense of direction and purpose in life”), and self-acceptance (e.g., “When I look at the story of my life, I am pleased with how things have turned out”). Participants are instructed to rate their agreement with each item on a six-point scale from one (strongly disagree) to six (strongly agree). The sum score for each component (range 7–42) and the total scale (range 42–252) will be calculated.

Emotional wellbeing is measured with the Satisfaction with Life Scale, which assesses a person’s satisfaction with his/her life as one global construct. The scale has five items rated on a seven-point Likert scale from one (strongly disagree) to seven (strongly agree). A sum score for the scale will be calculated (range 5–35) with higher scores indicating higher satisfaction with life [19]. Internal consistency, reliability and validity of the scale have been demonstrated [19, 20].

Depressive symptoms are assessed with the 20-item Centre for Epidemiologic studies Depression Scale (CES-D) [21]. The CES-D scale is a widely used self-report measure in population-based studies. Its reliability and validity has been demonstrated in heterogeneous samples [22]. Participant rate the frequency of each depressive symptom during the previous week. Each item is scored from zero (rarely or none of the time) to three (most or all of the time) with higher scores indicating more depressive symptoms (total score range 0–60). For participants with one missing item at most, the total score, ranging from 0 to 60, will be calculated. In the CES-D scale, the cut-off score indicating the presence of clinically important depressive symptoms in community-dwelling populations is 16 or more [23].

Quality of life is assessed with the 13-item version of the Older People’s Quality of Life questionnaire (OPQOL-brief). The scale includes items related to life overall as well as to more specific topics such as health, participation, social relationships and financial situation. The response options range from one (strongly disagree) to five (strongly agree) totaling to a sum score (range from 13 to 65; higher scores indicate higher quality of life). OPQOL-brief has shown high reliability and validity as a measure of overall quality of life [24]. In addition, participants are asked to evaluate the quality of their life and life satisfaction with the questions *“*Are you happy and satisfied with your life?” with response options: one (no), two (yes, occasionally) and three (yes, usually), and “How would you evaluate your life until now?” with response options: one (mostly unsatisfactory), two (occasionally unsatisfactory), three (mostly satisfactory). Self-rated health, an important aspect of quality of life, is assessed using a question on current general health with a five-point rating scale from one (very good) to five (very poor) [9]. Perceived stress in life is asked with a single question “Do you experience stress in your daily life?” with response options: one (no or hardly ever), two (yes, occasionally), and three (yes, often).

Perceived sense of autonomy in out-of-home participation, that is, the feeling of having control over the decision to go out whenever, wherever, and however one wants, is assessed with the ‘autonomy outdoors’ subscale of the Impact on Participation and Autonomy questionnaire (IPA) [25–27]. The IPA is a validated questionnaire designed to assess perceived autonomy and participation in various clinical and older populations. Participants are asked to rate perceived chances in visiting relatives and friends, making trips and traveling, spending leisure time, meeting other people, and living life the way they want. Response categories range from zero (very good) to four (very poor). A sum score (range 0–20) will be calculated, with a higher score indicating more restrictions in participation.

Self-reported functional status is assessed using a twelve-item questionnaire for Activities of Daily Living (ADL). Basic ADL functions include feeding, rising from or lying down on a bed, dressing, bathing, and toileting. Instrumental ADL functions include preparing a meal, shopping, light housekeeping tasks (e.g. doing the dishes), heavier housekeeping tasks (e.g. sweeping the floor), taking medicine, handling money, and using public transport. Participants are asked to rate the ability to perform each task: one (able without difficulty), two (able with some difficulty), three (able with a great deal of difficulty), four (unable without the help of another person), and five (unable even with help of another person) [27].

#### Physical activity

*Physical activity surveillance*. Willing participants wear two activity monitors continuously for 7 to 10 days during the time between the home interview and the assessments in the research center. The monitors to be worn are a tri-axial accelerometer (13-bit ±16 g capable of recording 10 days on one charge, UKK RM42, UKK Terveyspalvelut Oy, Tampere, Finland) and an ECG recorder which also includes a tri-axial accelerometer (14-bit ±16 g capable of recording 4 days on one charge, eMotion Faros 180, Bittium Corporation, Oulu, Finland). Both accelerometers are set to sample 100 samples per second. The accelerometer is attached to the anterior aspect of the mid-thigh of the dominant leg. The ECG recorder is attached with an adhesive strip that includes two electrodes 12 centimeters apart. The strip is attached either on the sternum or diagonally on the left side of the chest under the breast to ensure comfortable wear depending on the anatomy of the participant. The monitors are covered with a self-adhesive film for waterproofing. While wearing the monitors, participants complete a diary where they record occasions and reasons for removing the device(s), and their participation in any form of exercise other than walking, specifying the mode of exercise and the time. Participants wear the devices during the orthostatic test, and the cognitive and walking tests at the research center. Participants, who do not wear the activity monitors at home, are supplied with an accelerometer and ECG recorder with an electrode belt for their testing session at the research center.

*Accelerometry.* Movement and non-movement behaviors will be analyzed from both of the tri-axial accelerometer recordings [28]. Physical activity and sedentary behavior will be quantified as the amount of minutes spent at a particular activity intensity level (e.g. sedentary, light, moderate, vigorous). We will explore the usefulness of different bout durations, and gradations of intensity classification (e.g. a hundred possible intensity levels instead of the four conventionally used) in describing physical activity and sedentary time of older people [29, 30].

*Ambulatory ECG recording.* The ECG recorder produces a single-channel ECG recording at 250 samples per second. Heart beats (R-waves) will be identified from the recording using an automatic QRS detection algorithm [31], and day and night heart rate profile, cardiac autonomic modulation (heart rate variability) and physical activity intensity will be analyzed. We will also explore opportunities to identify previously undiagnosed cardiac arrhythmias, including atrial fibrillation from the ECG signal [32]. Validity of the heart rate and heart rate variability analysis will be ensured by filtering all technical and physiological artifacts from the RR interval time series. For heart rate variability analysis, a visual check and manual correction will be used in addition to automatic filtering.

*Numerical analysis for sedentary time and physical activity.* For both accelerometry and ECG the full and non-overlapping 24 h epochs starting from the first recorded mid-night will be used in the analyses. The data at both ends of the recording will be discarded. For accelerometry-based sedentary time and physical activity analysis the mean amplitude deviation of each 24 h epoch will be analyzed in one-minute non-overlapping epochs, and the mean daily physical activity will be reported as the mean of the 24 h and 7 day epochs [30, 33]. Similar approach will be utilized for heart rate, that is, the data will be reported for each 24 h epoch as one-minute non-overlapping mean heart rates.

Self-reported habitual physical activity is assessed using a single question [34] and using the Yale Physical Activity Survey for older adults (YAPS; [35]). A modified version of the multiple-choice question developed and validated by Grimby and Mattiasson-Nilo et al. [34, 36, 37] is used to assess the level of physical activity related to leisure-time, work and carrying out daily activities. Participants are asked to choose the description that best pictures their level of physical activity over the last year: one (hardly any activity, mostly sitting), two (light physical activity, such as light household tasks), three (moderate physical activity about 3 h a week: walking longer distances, cycling and domestic work), four (moderate physical activity at least 4 h a week or heavier physical activity 1 to 2 h a week), five (heavier physical activity or moderate exercise for at least 3 h a week), and six (competitive sports). This scale is feasible in older independent populations as it is easy and quick to use and also rates domestic activities. The test–retest reliability has been found to be fair [38].

The YAPS questionnaire includes a physical activity dimension sum index, which is the summation of five weighted subindices [35]. Participants are asked how many times they performed vigorous physical activity (weight 5) and leisure walking (weight 4) during the past month and the duration of each physical activity session. The frequency, duration score, and the weight of the respective activity will be multiplied. Additionally, participants are asked to estimate the duration of the time spent moving around (weight 3), standing (weight 2), and sitting (weight 1) on an average day in the past month. The duration scores will be multiplied with the weight to obtain a total score (range 0 to 137). Higher scores indicate higher physical activity. In addition, participants are asked to estimate whether their physical activity in the previous month differed from their activity in other annual seasons on a five-point scale from 1.3 (lot more) to 0.7 (lot less).

Unmet physical activity need is the feeling that one’s level of physical activity is inadequate, and thus distinct from the recommended amount of physical activity. Unmet physical activity need is the situation where a participant perceives to have no opportunities to increase their physical activity even though they are willing to do so. An existing question [39] is further developed to enable the assessment of the severity of unmet physical activity need. The new three-item questionnaire includes questions on willingness to increase physical activity, opportunities to increase physical activity, and current physical activity. A scoring method will be developed.

#### Resilience

The ten-item Connor-Davidson Resilience scale (CD-RISC) is used to measure psychological resilience, i.e. the ability to cope with adversity and positively adapt to changes in life. This unidimensional measure includes items such as “I’m able to adapt to change”, “I can achieve goals despite obstacles” and “I can handle unpleasant feelings”. Participants are asked to rate the extent to which the statements concord with their life using a five-point Likert scale ranging from zero (not true at all) to four (true nearly all the time). The total score ranges from 0 to 40 with higher values representing higher psychological resilience. The scale has shown good internal consistency and construct validity [40].

Heart rate variability quantifies the regulation of heart rate through the function of autonomic nervous system. Physiological variability and complexity are associated with the ability to adapt to external stressors [41]. ECG recordings of 24 h segments, a separate nocturnal sleep period and testing sessions at the research center (orthostatic test, cognitive tests and walking tests) will be analyzed in five-minute non-overlapping epochs. Time domain, frequency domain and non-linear measures of heart rate variability will be calculated [42]. Day and night heart rate profile, heart rate kinetics, cardiac autonomic modulation expressed in heart rate variability and complexity of RR interval time series will be explored as potential physiological indicators of resilience. Moreover, sedentary and physical activity behavior will be considered as a possible indicator of physical resilience.

Fatigability during cognitive and walking assessments assesses the level of fatigue in relation to the performance of well-defined activities. In conjunction with the walking tests, exercise tolerance and fatigability are assessed by repeated assessments of blood lactate concentration, and subjective ratings of perceived physical exertion and mental vitality, before the ten-meter walking tests and the six-minute walk test and immediately following the six-minute walk. Blood lactate concentrations are determined from capillary blood samples from a fingertip (BIOSEN Cline sport 2, EKF diagnostic). Participants are asked to rate their perceived physical exertion level with the Borg scale, ranging from six (no exertion) to twenty (completely exhausted) [43]. In addition, participants are asked to rate their mental vitality with a seven-point Likert scale, ranging from one (mentally exhausted) to seven (very alert and energetic) [adapted from [44]]. In addition, ECG is recorded during and for 2 min following the end of the six-minute walk while the participant sits quietly on a chair. Heart rate kinetics including heart rate increase and heart rate recovery will be assessed based on the ECG recording [45, 46]. In addition, the walking speed during the first and the final complete lap of the six-minute walk test will be compared to detect slowing of movement [47]. Similarly, for the cognitive assessments, results of a simple reaction time test before and after the cognitive test battery will be compared to detect slowing [adapted from [47, 48]].

Self-reported fatigability in daily life is assessed with the Situational Fatigue Scale [49]. The questionnaire assesses the level of fatigue related to 13 activities using a six-point Likert scale ranging from zero (not fatigued at all) to five (extremely fatigued). Scores will be summed to compute a total score (range 0–65), and the physical (playing a ball game, jogging, taking a walk, cleaning house) and mental (reading, watching TV, chatting, shopping, driving, hosting a social event, doing paperwork, meeting, attending a social activity) fatigue subscale scores. Higher scores indicate higher fatigability.

#### Environmental support and social engagement

Housing and living situation is assessed using several questions. The interviewer rates at the start of the home interview the type of dwelling (apartment block with or without elevator, row house, semi-detached or detached house). Participants are asked how many years they have lived in the same home, about their living situation (alone, with spouse, with children or grandchildren, with relatives, siblings or other people) and home ownership [27].

Perceived safety of the neighborhood is assessed with the question “Do you fear anything when moving through your neighborhood?”. If yes, participants are asked to specify their fear and to indicate whether the fear made them avoid moving in the neighborhood with response options 1) no, 2) yes, but only at night, and 3) yes.

Data on the use and perceptions of the neighborhood environment and beyond is gathered using an interactive internet-based softGIS or PP-GIS questionnaire (Maptionnaire, Mapita LTD, Helsinki, Finland) [50]. Participants are asked to locate, on a digital map, places where they have been physically active multiple times in the past month (outdoor sports facilities, indoor sports facilities, outdoor recreational areas). Similarly, participants are asked to locate other places motivating them to move outdoors in the neighborhood or beyond (e.g. nature, places to rest, routes, stores, services, and events) for multiple times in the past month. Follow-up questions specify the location, the frequency of visiting, and the mode of transportation used to get to the location. In addition, participants are asked whether they avoided any locations due to issues related to routes, safety, and other reasons in the past month, specifying the object and the occasions, and whether in general they perceive any environmental barriers that hinders their outdoor mobility (e.g. poor street conditions, hilliness, dangerous crossroads and lack of places to rest), specifying the barrier and the frequency of exposure. Finally, participants are asked to define their neighborhood as a polygon, and to locate one place (beyond their home) that gives them pleasure, specifying the object and the frequency of visiting the location. GIS will be used to construct an activity-space of each participant based on reported locations [adapted from [51]]. Features of the objectively assessed environment will be studied in participants’ individual activity-space and the neighborhood they defined as well as in spatial areas defined by network or circular buffers around participants’ home. Participants’ home addresses will be derived from the population register and geocoded in the GIS system. Features of participants’ home environment will be obtained from freely available digital maps (e.g. land use, street network, and services) and virtual audits, which utilize existing online virtual imagery resources (e.g. Google street view) to assess street level characteristics [adapted from [52, 53]].

Socioeconomic status. Education is assessed by asking participants to report the total number of years of education they completed, and by choosing their highest educational attainment from a list, which includes seven alternatives: one (less than primary school), two (primary school), three (middle school or folk high school), four (vocational school), five (secondary school), six (high school), and seven (university degree) [54]. Occupation is assessed by asking participants to report their longest-held occupation and their most recent occupation. The resulting occupations will be classified according to the Statistics Finland’s Classification of Occupations [55], which is based on the International Standard Classification of Occupations ISCO-08 by the International Labour Organization. Participants are asked to rate their self-perceived financial situation on a five-point scale ranging from one (very good) to five (very poor).

Feeling of loneliness is asked with a single question: “How often do you feel lonely?” The response options are rated on a four-point scale: one (very rarely/never), two (rarely), three (often), and four (almost always) [56]. Social relationships and contacts are assessed with questions relating to the participants’ marital status and the frequency of contacts with children and other relatives, close friends, and other acquaintances. The response options for the frequency of contacts are rated as follows: one (daily), two (weekly), three (monthly), four (a few times a year), five (rarely or not at all), and six (not having any children or other relatives/friends/acquaintances) [27]. Moreover, participants are asked whether they have someone with whom they regularly run errands or enjoy the outdoors.

Provision of social support is assessed with a single questions related to volunteer work and informal care provision. Volunteer work is asked with the question “Do you volunteer for some organization, municipal, congregation, etc.?” with response options: one (daily or almost daily), two (approximately once a week), three (approximately once a month), four (few times a year), five (rarely), and six (not at all). Informal care provision is asked with the question “Do you take care of another person needing assistance in daily life due to illnesses or disabilities?” [27]. Caregivers are also asked whether they live in the same home as the care receiver (yes/no) and how often care is generally provided. The response options for the latter question are: one (almost round-the-clock), two (daily), three (few times a week), four (once a week), five (two to three times a month), and six (once a month or less frequently).

Current most important personal hobbies are asked with a single open-ended item question “What are your most important hobbies?” Participants can report as many hobbies as they want.

Perceived age discrimination is assessed with a single item “During the previous year, have you experienced that you have been discriminated or placed in a disadvantaged position compared to other people due to your age (e.g. in offices, health care organizations, or in everyday life)?” The response options are: one (not at all), two (only a little), three (to some extent), four (quite a lot), and five (very much) [adapted from [57]].

#### Physical and sensory function

Maximal isometric handgrip strength is measured on dominant side both in the home interview and in the research center. Handgrip strength has been shown to be a good indicator of general functional status [58]. *During the home interview*, handgrip strength is measured with a hand-held adjustable dynamometer (Jamar Plus digital hand dynamometer, Patterson Medical, Cedarburg, WI, USA), and expressed in kg [58]. The measurement is done in a seated position with the elbow flexed in an angle of approximately 90 degrees. After a practice trial, three to five short maximal contractions with 30 s of rest in-between are conducted. *In the research center,* maximal isometric handgrip strength is measured in a sitting position using an adjustable dynamometer chair (Faculty of Sport and Health Sciences, University of Jyvaskyla, Jyväskylä, Finland), and expressed in Newton [34]. A dynamometer is fixed to the arm of the chair. After a practice trial, the test is performed at least three times until no further improvement occurs, with an inter-trial rest period of 1 min. In addition, maximal isometric knee extension strength is measured in a sitting position using an adjustable dynamometer chair (Metitur LTD, Jyväskylä, Finland), and expressed in Newton [34]. Knee extension strength of the dominant leg is measured at a knee angle of 60 degrees from the fully extended leg towards flexion. The ankle is attached to a strain-gauge system. After a practice trial, the test is performed at least three times until no further improvement occurs, with an inter-trial rest period of 1 min. In the research center, the calibration of the dynamometers is checked daily before use. During each maximal contraction of 2 to 3 seconds, participants are strongly encouraged to exhibit the best possible force. For each test, the best result will be used as the measure of maximal strength in the analyses. The test-retest reliability of the handgrip and knee extension strength test with a 2-week interval has been found to be good in our research center [34].

Reaction time and sensory motor speed are assessed first with a simple finger movement task, followed by a complex finger movement task [59]. The seated participant holds index finger of the dominant hand resting on the rest button in the middle. The participant will move the finger as soon as possible to the button closest to the light, when it switches on. First, the simple reaction time test is conducted and then a more complex test, where any of the seven lights switches on randomly. Reaction time and movement time are measured in milliseconds. The simple and complex tasks are each repeated 12 times. The average times of the final five correctly performed tasks will be used as the result.

Lower-extremity physical performance is objectively assessed in the participant’s home by the Short Physical Performance Battery (SPPB) [60–62]. The battery comprises tests on standing balance, walking speed over a 3-m distance, and the ability to rise from a chair. Each task is rated from zero to four points according to established cut-off points [61, 62], higher scores indicating better performance. Participants unable to perform a test due to mobility-related limitations will be assigned a score of zero for each respective test. A sum score will be calculated (range 0–12), when at least two tests are completed.

Ten-meter walking speed is assessed in the laboratory corridor. The time to walk ten meters with habitual and maximal speed is measured using photocells (Faculty of Sport and Health Sciences, University of Jyvaskyla, Jyväskylä, Finland) and a hand-held stopwatch (for cohort comparisons) and walking speed expressed in m/s will be calculated. On their first walk, participants are instructed to walk at their habitual speed, that is, the speed they would use when running errands [63]. On the second walk, participants are instructed to walk as fast as possible, without compromising safety, for the maximal test [64]. In both tests, five meters is allowed for acceleration, and the walking stops well past the finish line. Participants wear walking shoes or sneakers and are allowed to use a walking aid if needed. The test-retest precision of the maximal walking test with a one- to two-week interval has been shown to be good in our research center [65].

A modified six-minute walking test with a usual walking speed is used to assess walking performance, exercise tolerance and cardiovascular response to exercise [47, 66]. Usual, self-paced walking speed rather than maximal speed ensures the safety of the older participants and promotes continuous walking performance over the duration of the test [47]. Participants are allowed to use a walking aid, if needed. The test is performed in an indoor corridor. Traffic cones are placed at both ends of the course 19.66 m apart, and tape indicates the bend with a 0.30 m radius, resulting in a 40-m lap. Photocells (Faculty of Sport and Health Sciences, University of Jyvaskyla, Jyväskylä, Finland) are placed 18.0 m apart and 0.83 m from each end to record lap times. The total distance walked by participants in 6 min is measured.

*During the six-minute walk test,* gait characteristics are assessed using wearable sensors. Participants are asked to wear five sensors (NGIMU, x-io Technologie Limited, UK) that sample three-dimensional accelerations, gyrations and magnetic field at 400 samples per second during the gait assessment. Sensors are attached to both legs with elastic Velcro straps: above the lateral malleolus and on the anterior aspect of the mid-thigh (co-localized with the taped-on accelerometer used to record physical activity). The fifth sensor is worn on the mid back line at the L4 to S1 level depending on anatomy of the participant. Step or stride rate [67], stance and swing duration [68], and multiscale entropy [67, 69] will be assessed from each of the applicable sensors, and from the thigh-worn accelerometer and the chest-worn accelerometer. Furthermore, we will explore whether age- and functional capacity -related changes in gait can be detected. In addition, gait characteristics will be identified *based on multiple day accelerometry recordings*, enabling comparison with laboratory-based gait entropy assessment. Bouts of continuous walking will be identified from both accelerometer recordings based on activity intensity, and any bouts of at least 1 min will be included in evaluating gait dynamics. Multiscale entropy will be analyzed from each of the gait bouts, and the mean of all bouts will be reported [69, 70].

Hearing acuity. Participants are asked about use of hearing aids. Self-rated hearing is assessed using a question “How is your hearing?”, and it is answered by choosing a number along a continuum between zero (very poor) and ten (very good). Participants using hearing aids evaluate their hearing with and without the aid. In addition, participants are asked whether they are able to hear in a normal conversation with three or more persons with response options: one (yes, without difficulty), two (yes, with some difficulty), three (yes, with a great deal of difficulty), and four (no, not at all). Pure-tone screening audiometry (Oscilla USB-330, Inmedico A/S, Denmark) and Peltor noise reducing headphones with a noise reduction rating of 21 dB are used to measure pure-tone air-conducted hearing thresholds [71]. Hearing thresholds are estimated using the automatic Hughson-Westlake protocol at the frequencies of 0.125, 0.25, 0.5,1, 2, 4, and 8 kHz. Both ears are measured separately. The maximum sound intensity is 90 dB. If the participant does not hear at an intensity of 90 dB, 100 dB is recorded as the hearing threshold.

Visual acuity. Self-rated vision is assessed by asking about the use of spectacles, and if yes, what kind of spectacles with response options: one (reading glasses), two (glasses for farsightedness), three (glasses for reading and farsightedness), four (multifocal glasses), and five (other). Near vision is assessed by asking whether the participant is able to read normal newspaper text. Far vision is assessed by asking whether the participant is able to watch television from a normal watching distance (three meters). The response options are: one (yes, without difficulty), two (yes, with some difficulty), three (yes, with a great deal of difficulty), and four (no, not at all). Binocular visual acuity is assessed first without and then with the participant’s own spectacles. Illuminated Landolt ring chart (Oculus 4512) at a 5 meter distance is used for assessment. The Landolt rings are widely accepted as the standard of reference in measuring distance visual acuity [72].

Respiratory function is assessed with spirometry (Medikro Pro spirometer, Medikro Oy, Kuopio, Finland) in a standing position with a nose clip. Firstly, vital capacity (VC) maneuver is performed two to four times. Participants inhale maximally and exhale into a flow transducer of the spirometer and continue until their lungs are completely empty. Secondly, forced vital capacity (FVC) maneuver is performed at least two times. Participants inhale maximally and exhale fast and forcefully into the flow transducer and continue until their lungs are completely empty. Both VC and FVC maneuvers are discontinued once they meet the criteria of the ATS/ERS Taskforce [73] or when a total of eight exhalations is reached. The highest volume of VC, FVC and the forced expiratory volume in 1 second (FEV1) are recorded in liters, and the peak expiratory flow (PEF) is recorded in liters/second.

#### Cognitive capacity

Cognitive impairment is assessed with the Mini-Mental State Examination (MMSE) [74]. The MMSE contains 19 items and scores range from 0 to 30. For those participants who are not able to do one or more parts of the MMSE questionnaire due to issues unrelated to cognition, e.g. blindness, the total score is scaled. Participants unable to write are allowed to dictate the sentence. No reductions in scoring will be made because of this.

The Trail Making Test (TMT) is a widely used paper-and-pencil test to assess visual search, scanning, speed of processing, cognitive flexibility, and executive functions [75]. The test consists of two parts. In TMT-A, 25 encircled numbers are distributed on a sheet of paper, and the aim is to connect the numbers with a line as fast as possible (1–2–3-4-5, etc.). In TMT-B, the task is similar, except that the person must alternate between numbers and letters (1-A-2-B-3-C, etc.). If the participant makes an error, the examiner returns him/her to the last correct response immediately. The time required to complete each task is the participant’s score. The test result is disqualified if the time limit of 300 s is exceeded or four or more errors occur [76]. The TMT-A part assesses mainly visual search and motor speed skills, whereas TMT-B measures attentional control and cognitive flexibility [77–79]. B/A ratio of performance has been associated with executive functions [80].

Digit span test is a popular measure of auditory short-term memory, requiring the verbal recall of forward and backward number series, which the examiner says to the participant [81, 82]. The test starts with forward-series of four digits, and the string gets longer up to eight digits, until the participant fails twice. Then the same procedure is repeated, but the digits must be repeated backwards (from two to seven digit series). The score is the number of correctly repeated digit spans in both forward and backward tests (maximum 17). The forward digit span relies on the phonological loop of working memory, whereas the backward digit span also engages the central executive component [83].

Digit symbol coding task is a paper-and-pencil test measuring processing speed and short-term visual memory (The Wechsler Adult Intelligence Scale-Revised) [84] . The participant has to draw correct symbols below their equivalent numbers by using a number-to-symbol coding key. Time limit of the task is 90 s, and the score is the number of correct symbols in correct order (maximum 65). Several abilities are needed to perform well in the task, and scores have been found to decline steeply with age [85, 86].

Phonemic verbal fluency is measured with a modified, version of Word Fluency [82, 87]. In the test, participants are instructed to name as many Finnish words as possible, starting with the letter K, during 3 min (instead of the original 5 min). The examiner writes down the words said by the participant, and the score is the number of acceptable words (including a limited number of names). Verbal fluency test measures semantic memory and verbal ability, and it is a sensitive indicator of brain dysfunction, because the task requires both clustering (certain phonemic category) and the ability to shift efficiently to a new strategy [88].

#### Physical health

Information on self-reported physician diagnosed chronic diseases is collected during the home interview by prompting participants with ten categories of chronic conditions and then specifying the condition. Listed are: respiratory conditions (asthma, chronic obstructive pulmonary disease, chronic bronchitis, other), cardiac conditions (myocardial infarction, coronary heart disease, heart failure, atrial fibrillation or other arrhythmias, other), vascular conditions (hypertension, thrombosis or intermittent claudication, other), cerebrovascular condition or brain injury (stroke or cerebral infarction, brain injury, other), musculoskeletal condition (rheumatic arthritis, osteoarthritis, chronic back pain or problems, chronic neck pain or problems, osteoporosis, other), visual or auditory impairment (cataract, not surgically repaired, glaucoma, macular degeneration, hearing disorder, hearing injury or other hearing debilitating condition), diabetes mellitus, malignant cancer, neurological condition (Parkinson’s disease, Alzheimer or dementia, epilepsy, other), and depression. Furthermore, an open-ended question about any other physician diagnosed chronic conditions is used. A nurse will categorize the other conditions listed, after which a morbidity index will be calculated similar to one previously used [27]. For some conditions, follow-up questions are asked to ensure safe participation in the physical tests and to form the basis for the clinical examination in the research center. These questions are: pain and symptoms in case of a respiratory condition and/or a cardiac condition, type of surgery in case of coronary heart disease, pacemaker in case of arrhythmias, the type (I or II) and use of medication in case of diabetes mellitus, and the phase of cancer treatment in case of cancer. Finally, questions concerning the presence of severe pain in the back, knees, hips or other locations which hinder daily life, fractures and hospital admissions in the previous year are queried.

Participants are asked to report all medication prescribed by a physician in the postal questionnaire and to indicate whether they use the medication regularly or occasionally. Medication will be categorized [89] and medication potentially affecting cardiac autonomic modulation will be identified.

Clinical health examinations by a research nurse and a physician are organized in the research center for assessing health status and ensuring safety of participants during the physical assessments. The clinical examination includes a review of self-reported chronic conditions and medical prescriptions, current symptoms, blood samples, a resting ECG, and an orthostatic test. If necessary, also glucose levels and oxygen saturation in the blood can be measured by the physician. Because the functional assessments are not more straining than daily life, exclusion from tests is considered case by case.

Blood samples are drawn prior to the health examination in the research center. C-reactive protein levels are determined to ensure safe participation in the physical assessments. Other blood markers include a small blood count, total cholesterol, HDL and LDL cholesterol, and vitamin D. Blood samples will be destroyed once these markers have been determined.

Resting ECG is recorded for ensuring safety of participating in the functional assessments in the research center. Standard 12-lead ECG is recorded after a minimum of 5 min of supine rest and electronically stored (CardioSoft V6.73, GE Healthcare, Chicago, IL, USA). Following the supine rest, an active orthostatic test is performed to assess cardiovascular responses to orthostasis and to study orthostatic tolerance to ensure safety of participants during physical assessments. Having lied down for at least 10 min, the participant stands up and then quietly stands for 6 min. Possible clinical symptoms, such as light-headedness, dizziness or syncope are recorded [90]. Blood pressure is measured during supine rest 1 min before standing up, and again immediately after standing up, and three and 5 min after standing up. Orthostatic heart rate and heart rate variability is assessed using continuous ECG recording starting at 5 min before standing up until 6 min after standing up.

Arterial stiffness, which is an important predictor of cardiovascular disease [91], is measured by the method of pulse wave analysis using the Diagnostic Station DS20 (Schiller AG, Baar, Switzerland). The DS20 is a non-invasive cuff-based device capturing brachial blood pressure and pulse wave forms to estimate the central aortic hemodynamics and pulse wave velocity. After resting in a seated position for at least 10 min to ensure hemodynamic stability, measurements are performed on the dominant arm with the arm positioned on a table so that the middle of the cuff on the upper arm is at the level of the heart. Participants are instructed to refrain from talking and to sit with their back resting against the chair backrest, their feet flat on the floor, and their legs uncrossed. Three measurements per participant with a one-minute rest in between measurements are performed. Each measurement starts with a recording of brachial blood pressure followed by a pulse wave recording with the cuff inflated at the diastolic blood pressure level. Ten stable consecutive pulses are filtered and averaged by the device to calculate the central aortic pulse wave. Evaluation of shape and amplitude of the wave results in the following indirect measures of arterial stiffness (among others): pulse wave velocity, augmentation pressure, and augmentation index [92]. Pulse wave velocity is an estimate of the speed of the pressure wave traveling along the aortic and aorto-iliac pathway [93]. Augmentation pressure and augmentations index are both derived from the ascent of the aortic pressure waveform in late systole attributed to the early return of pulse wave reflection from peripheral sites [94, 95]. Due to heart rate dependency augmentation index is normalized at a heart rate of 75 beats per minute [96]. Blood pressure is measured in conjunction with the arterial stiffness assessment.

Objective anthropometric measurements are taken in the research center. Body height is measured while the participant is standing in an upright position on a stadiometer. Two consecutive measures are taken and the final result, recorded to the nearest 0.5 cm, is the mean of the two values. Body weight is measured in light clothing while the participant stands on an electric scale (Seca, Hamburg, Germany). Weight is recorded to the nearest 0.1 kg. Body mass index will be calculated as weight in kilograms divided by height squared in meters (kg/m^2^). Waist circumference is measured according to the instructions of the World Health Organization [97]. The measurement site is the midpoint between the lowest palpable rib and the top of the iliac crest [97]. Three consecutive measures are taken from the bare skin at the end of an exhalation. The mean of the three values will be recorded as the final result. Multi-frequency bioelectrical impedance measurement (InBody 720, Biospace, Seoul, Korea) provides information on body composition, that is, e.g. body fat mass, lean body mass and its distribution in the body. Measurements are performed according to the instructions of the manufacturer with participants wearing light clothing and standing barefoot on the device and holding the handles in both hands. Cases of non-removable metal jewelry or metal in the body are recorded. Participants with a pacemaker are excluded from the bioimpedance measurement.

Physical frailty phenotype will be determined according to the criteria by Fried et al. [98]. Physical frailty phenotype indicators are: self-reported unintentional weight loss of > 5 kg in the past year (question 1 from SCREEN II-AB questionnaire [99]), exhaustion (questions seven and twenty of CES-D Scale [21]), and low physical activity (self-reporting only light physical activity or less [36, 100]), weakness (lowest quintile of handgrip strength from hand-held dynamometer in their own sex and age group), slowness (lowest quintile of three-meter normal walking speed in their own sex and age group). Frailty status is defined as no frailty (no indicators present), pre-frailty (one to two indicators), and frailty (≥3 indicators) [98].

#### Health behavior and health literacy

The average alcohol consumption is assessed separately for beer and cider, wine, and distilled beverages using five-point scales. For beer and cider, the response options range from zero (not at all) to four (more than 12 bottles a week), for wine, from zero (not at all) to four (more than two bottles a week), and for distilled liquor, from zero (not at all) to four (more than four bottles a month). In addition, we ask participants whether other people have been worried about their alcohol consumption or whether others have suggested drinking less. The response options are: one (never), two (yes, but not during the last year), and three (yes, during the last year). Smoking history is assessed by asking whether participants smoke or have smoked daily or almost daily at least for a year. Past smokers are asked to specify the age at smoking cessation [27].

Perceived own role in health behavior is assessed with three structured questions. Participants are asked whether they believe they can contribute to maintaining their health with response options: one (yes, I believe my contribution is very important), two (yes, I think my contribution matters), and three (no, I don’t think my contribution matters). Participants are asked whether they do something for maintaining or promoting their health, and to specify their act from a seven-item list: to exercise or to be physically active, to eat healthy, to try to quit smoking or decrease smoking, to take care not to drink too much alcohol, to take care not to work too much, to ensure sufficient sleep, and something else.

Nutritional status and habits are assessed using the abbreviated questionnaire Seniors in the Community: Risk evaluation for eating and nutrition (SCREEN II-AB; The SCREEN II questionnaire is the copyright of Dr. Heather Keller). It is a valid and a reliable measure for detecting older people at risk for impaired nutritional status [99, 101]. The questionnaire consists of eight items assessing food habits and risk factors for malnutrition. The response scores of each item ranges between zero and four (items on fruit and vegetable servings, fluid intake, company with meals, and meal preparation) or between zero and eight (items on weight change, skipping meals, appetite, and difficulty swallowing). A sum score will be calculated (range 0–48), with lower scores indicating higher nutritional risk [99].

Health literacy is measured with the short version of the European Health Literacy Survey Questionnaire (HLS-EU-Q16). The short version of the HLS-EU-Q includes 16 items, covering the domains of health care, disease prevention and health promotion [102]. Participants are asked to rate the perceived difficulty in different tasks of accessing, understanding, appraising and applying health information. The response options are: one (very difficult), two (fairy difficult), three (fairly easy), and four (very easy). A general index of health literacy will be calculated for respondents with at least 80% of the health literacy questions completed using the formula [103]:

$$ Index=\left( mean-1\right)\ast \left(\frac{50}{3}\right) $$

The final index score will range from 0 to 50, with higher scores representing better health literacy. The score can be divided into inadequate (0–25), problematic (> 25–33), sufficient (> 33–42) and excellent (> 42–50) health literacy [104].

#### Mobility

Life-space mobility reflects actual mobility performance in daily life and is assessed with the University of Alabama at Birmingham Study of Aging Life-Space Assessment (LSA) [105, 106], which has good test-retest reliability. The LSA comprises 15 items and assesses mobility through the different life-space levels (bedroom, other rooms, outside home, neighborhood, town, beyond town), which the participant reports having moved through during the 4 weeks preceding the assessment. For each life-space level, participants are asked how many days a week they attained that level and whether they needed help from another person or from assistive devices. A composite score will be calculated (range 0–120), reflecting the spatial area through which a person moves, the frequency of movement and the need for assistance. Higher scores indicate greater life-space mobility.

The use of different transportation modes is assessed by asking how often participants drive a car, travel by car as a passenger, use public transportation such as a bus or a train, and use taxi or Special Transportation Services [27]. The response options are: one (daily or almost daily), two (a few times a week), three (a few times a month), four (a few times a year), five (less than once a year), and six (never). Participants who answer that they never drive a car are asked to specify whether they have never driven a car or whether they have stopped driving a car.

Self-reported mobility limitations are assessed as perceived difficulty in walking 500 m and two kilometers, and mounting a flight of stairs [107, 108]. The response options are: one (able to manage without difficulty), two (able to manage with some difficulty), three (able to manage with great deal of difficulty), four (able to manage only with help of another person), and five (unable to manage even with help). For each task, those who report being able to manage without difficulty are asked whether they have modified their way of performing the task (mobility task modification) [107]. The question is “Have you noticed any of the following changes in your ability to walk 2 km/500 m/mount a flight of stairs?” The potential modifications are: having slowed down the pace, resting in the middle of performing the task, using an assistive device, and having reduced the frequency of performing the task, and the response options are: one (yes) and two (no). In addition, we enquire whether the person has given up doing the task or if the person experiences tiredness when doing the task. For each task, there is an additional open-ended question about other changes in the ability to perform the task. The questions regarding task modification identify older people in an intermediate phase between intact mobility and manifest mobility limitation [108]. In addition, participants are asked whether they use the assistive devices listed: walking stick, crutches, Nordic walking sticks, rollator, kicksled/kickcycle, wheelchair, electric scooter, other.

Fear of falling is assessed by the question “Are you afraid of falling?” with response options: one (never), two (occasionally), three (often), and four (constantly) [27]. Fall history is assessed using the question “Have you fallen or slipped during the previous year?” with response options: one (no), two (yes, outdoors), three (yes, indoors), four (yes, both indoors and outdoors). Fallers are asked whether they have fallen once or multiple times, and whether any fall resulted in an injury, which required treatment by a physician.

#### Other aspects

The home interview and the laboratory assessments are completed by asking participants whether they want to share any thoughts and feelings about the interview or assessments with open-ended question (debriefing). After the home interview, participants are also asked whether something out of the ordinary has recently happened in their lives that they feel has influenced their answers. Participants can freely share their thoughts on such occasions. If necessary, messages are registered.

Information on age and sex are drawn from the population register. Life years will be calculated in 2028, approximately 10 years after the initial assessments. Death dates will be requested from the Population Register. Survival time will be calculated from the day of the home interview to the day the participant died [9]. Participants not known to have died or lost to the follow-up will be given a survival time of 10 years.

### Data management and quality assurance

#### Data management

The University of Jyvaskyla fully owns the research data. Consent forms and paper questionnaires are stored in locked cabinets in a project researcher’s office. Digital data are stored on computer drives of the Information Management Center of the University of Jyvaskyla. All datasets are pseudonymized. The research data are managed by AGNES research team members appointed for this task. The key identifying participants is stored separately from data files and only accessible to designated research team members. In 2023, we will re-evaluate the need to store the identification key. For all participants, once vital status is checked in 2028, the identification key will be destroyed. All research team members can use the pseudonymized datasets for research and teaching for the time when they are employees of University of Jyvaskyla.

#### Quality assurance

The interviewers and testers participated in a training course on interviewing techniques suitable for older people, the scales and tests, study ethics and safety as well as practice interviews and testing sessions. Manuals of operations have been prepared prior to the start of the study (September 2017) and will be updated if necessary. Computer assisted interviewing techniques are used, whenever possible. Testing sessions are periodically observed. We monitor all incoming data for consistency and organize periodic meetings with research staff.

### Statistical analyses

Non-respondent analyses will be conducted to determine differences between participants in different parts of the AGNES study and those not participating in the respective parts in order to establish potential selection bias.

Full information maximum likelihood (FIML) estimation under the assumption of data missing at random (MAR) will be used in analyzing incomplete data. Multiple imputation methods will be used to complete the dataset for efficient and valid analyses. In sum score calculations, limited numbers of missing items (e.g. one or two), will be imputed with the participant’s mean of completed items (scaling), while in some cases best available information may be used to replace missing values.

The study data to be collected will consist of a cross-sectional dataset. Path modeling and structural equation modeling will be used for multilevel analyses of environmental and individual characteristics relative to the study outcomes. The cross-sectional data will be analyzed with Bayesian methodology permitting better control of expectations generated by previous research, and interpretability of the results. Markedly non-normally distributed data will be transformed prior to inferential comparisons. For variables that cannot be successfully transformed, nonparametric methods will be used. Categorical variables will be analyzed with logistic regression or categorical response models. If the expected frequencies are too small for asymptotic assumptions, exact testing techniques will be used.

### Data reporting

Results will be primarily reported in articles published in established scientific journals. In all publications, results from the observational study will be reported following the STROBE criteria (Strengthening the Reporting of Observational studies in Epidemiology; [109]).

## Discussion

The current study will produce new knowledge regarding the prerequisites, determinants and modifiers and consequences of active aging. It will also report on whether physical and cognitive functioning of later born cohorts of 75- and 80-years-old people has changed. The study will have high multidisciplinary impact by providing us with novel information on determinants and modifiers of the active aging pathway and the dimensions of active aging. In the future, the cohort will also form an important platform for future longitudinal studies either serving as the baseline for them, or else the participants may be recruited from AGNES to supplementary detailed satellite studies on specific, well-defined groups.

The current study is cross-sectional, and does not provide knowledge on the temporal order of events and causality remains unclear. However, an innovative cross-sectional dataset provides an opportunity for emergence of novel creative study hypothesis and theories to be tested in future studies, and is thus a key to regeneration of science. The novel assessment method of active aging and other novel indicators and biomarkers of physical activity and resilience and the multidisciplinarity of the current study will propel forward the research on aging and functioning.

## Abbreviations

ADL: Activities of daily living
CES-D: Centre for Epidemiologic studies Depression Scale
ECG: Electrocardiogram
FEV1: Forced expiratory volume in one second
FIML: Full information maximum likelihood
FVC: Forced vital capacity
GIS: Geographic information system
HLS-EU-Q16: European Health Literacy Survey Questionnaire
IADL: Instrumental activities of daily living
IPA: Impact on Participation and Autonomy questionnaire
LSA: University of Alabama at Birmingham Study of Aging Life-Space Assessment
MAR: Missing at random
MMSE: Mini-Mental State Examination
OPQOL: Older People’s Quality of Life questionnaire
PEF: Peak expiratory flow
SCREEN: Seniors in the Community: Risk Evaluation for Eating and Nutrition
SPPB: Short Physical Performance Battery
STROBE: Strengthening the Reporting of Observational studies in Epidemiology
TMT: Trail Making Test
UJACAS: University of Jyvaskyla Active Aging Scale
VC: Vital capacity
